# Spo0A Suppresses *sin* Locus Expression in Clostridioides difficile

**DOI:** 10.1128/mSphere.00963-20

**Published:** 2020-11-04

**Authors:** Babita Adhikari Dhungel, Revathi Govind

**Affiliations:** a Division of Biology, Kansas State University, Manhattan, Kansas, USA; University of Iowa

**Keywords:** *Clostridioides difficile*, *C. difficile*, SinR, Spo0A, gene regulation, virulence gene regulation

## Abstract

Clostridioides difficile is the leading cause of nosocomial infection and is the causative agent of antibiotic-associated diarrhea. The severity of the disease is directly associated with toxin production, and spores are responsible for the transmission and persistence of the organism. Previously, we characterized *sin* locus regulators SinR and SinR′ (we renamed it SinI), where SinR is the regulator of toxin production and sporulation. The SinI regulator acts as its antagonist. In Bacillus subtilis, Spo0A, the master regulator of sporulation, controls SinR by regulating the expression of its antagonist, *sinI*. However, the role of Spo0A in the expression of *sinR* and *sinI* in C. difficile had not yet been reported. In this study, we tested *spo0A* mutants in three different C. difficile strains, R20291, UK1, and JIR8094, to understand the role of Spo0A in *sin* locus expression. Western blot analysis revealed that *spo0A* mutants had increased SinR levels. Quantitative reverse transcription-PCR (qRT-PCR) analysis of its expression further supported these data. By carrying out genetic and biochemical assays, we show that Spo0A can bind to the upstream region of this locus to regulates its expression. This study provides vital information that Spo0A regulates the *sin* locus, which controls critical pathogenic traits such as sporulation, toxin production, and motility in C. difficile.

**IMPORTANCE** Clostridioides difficile is the leading cause of antibiotic-associated diarrheal disease in the United States. During infection, C. difficile spores germinate, and the vegetative bacterial cells produce toxins that damage host tissue. In C. difficile, the *sin* locus is known to regulate both sporulation and toxin production. In this study, we show that Spo0A, the master regulator of sporulation, controls *sin* locus expression. Results from our study suggest that Spo0A directly regulates the expression of this locus by binding to its upstream DNA region. This observation adds new detail to the gene regulatory network that connects sporulation and toxin production in this pathogen.

## Abstract (precis)

Clostridioides difficile is the leading cause of antibiotic-associated diarrheal disease in the United States. During infection, C. difficile spores germinate, and the vegetative bacterial cells produce toxins that damage host tissue. In C. difficile, the *sin* locus is known to regulate both sporulation and toxin production. In this study, we show that Spo0A, the master regulator of sporulation, controls *sin* locus expression. Results from our study suggest that Spo0A directly regulates the expression of this locus by binding to its upstream DNA region. This observation adds new detail to the gene regulatory network that connects sporulation and toxin production in this pathogen.

## INTRODUCTION

Clostridioides difficile is a Gram-positive, anaerobic bacillus and is the principal causative agent of antibiotic-associated diarrhea and pseudomembranous colitis (1–3). Antibiotic use is the primary risk factor for the development of C. difficile-associated disease because it disrupts normal protective gut flora and provides a favorable environment for C. difficile to colonize the colon. Two major pathogenic traits of C. difficile are toxins (toxins A and B) and spores (3–5). Deaths related to C. difficile increased by 400% between 2000 and 2007, in part because of the emergence of more aggressive C. difficile strains (6, 7). Robust sporulation and toxin production were suspected of contributing to widespread C. difficile infections associated with these highly virulent strains (8–14). How C. difficile triggers toxin production and sporulation in the intestinal environment is only beginning to be understood.

We recently reported the identification and characterization of master regulator SinR in C. difficile, which was found to regulate sporulation, toxin production, and motility (15). SinR in the Gram-positive model organism Bacillus subtilis is well characterized and is known to regulate multiple pathways, including sporulation, competence, motility, and biofilm formation (16–18). In B. subtilis, SinR is encoded by the downstream gene of the two-gene operon called the *sin* (*s*porulation *in*hibition) locus, and its transcription is driven from two promoters. The second gene in the operon, *sinR*, is transcribed by an internal promoter and is constitutively expressed. SinR represses the first committed (stage II) genes in the sporulation pathway (17). The promoter upstream of the operon is activated by phosphorylated Spo0A, leading to the expression of *sinI*, along with *sinR*. The SinI protein binds and inhibits the DNA binding activity of SinR (19–21). The combined effect of positive regulation by phosphorylated SpoA (Spo0A∼P) and the inactivation of the negative regulator SinR activates the sporulation pathway. In C. difficile also, the *sin* locus is a two-gene operon and encodes SinR and SinI (previously SinR′). In our initial characterization of the C. difficile *sin* locus, we showed that disruption of the *sin* locus (absence of both SinR and SinI) resulted in an asporogenic, less toxic, and less motile phenotype (15). Another study, which reports that C. difficile *sin* locus suppresses biofilm formation, corroborates our finding (22). Further investigation showed that among the two regulators, SinR positively influences sporulation, toxin production, and motility, while SinI acts as an antagonist to SinR and controls its activity (15). Since the *sin* locus has a role in regulating various pathogenic traits in C. difficile, understanding the regulation of its expression is important. Earlier, we showed that disruption of the first gene in the operon, *sinR*, affects transcription of both *sinR* and the downstream gene *sinI*. This observation led to the assumption that unlike B. subtilis, the C. difficile *sin* locus is transcribed from a single upstream promoter. Real-time reverse transcription-PCR (RT-PCR) analysis of the cells grown *in vitro* showed *sin* locus expression at the 10-h time point, indicating its tight regulation (15). From various gene expression data, we can observe that mutations in *sigH* and *spo0A* positively influence the expression of *sinRI* (23–26) and mutations in *tcdR* act to downregulate their expression (27). We have also demonstrated that CodY can directly bind to the *sin* locus upstream DNA to transcriptionally repress its expression (15). In the same line of investigation, in this study, we discovered that Spo0A, the sporulation master regulator, represses *sin* locus expression. The effect was directly caused by the specific binding of Spo0A to the promoter region upstream of the locus.

## RESULTS

### An elevated level of SinR is present in the *spo0A* mutant.

**FIG 1:**
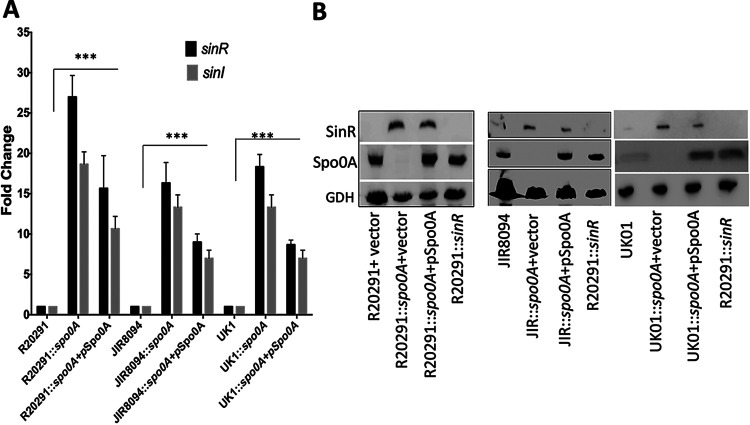
In the absence of Spo0A, C. difficile produces elevated levels of SinR. (A) qRT-PCR results of *sin* locus transcripts in C. difficile strains collected at the 10-h time point. The representative results from three independent experiments are shown. The asterisks (***) indicate statistical difference at *P* < 0.005. (B) Western blot analysis of parent strains (R20291, JIR8094, and UK01) and their respective *spo0A* mutants and complemented strains using SinR- and Spo0A-specific antibodies, demonstrating upregulated SinR in the absence of Spo0A. The *sinR* mutant served as a negative control. Glutamate dehydrogenase (GDH) detection using anti-GDH antibodies was used as loading control.

**FIG 2:**
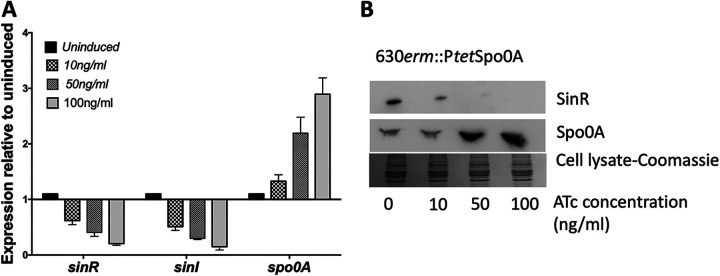
Spo0A suppresses *sin* locus expression in the 630*erm*::P*tet*Spo0A strain in a dose-dependent manner. (A) qRT-PCR analysis of *sinR*, *sinI*, and *spo0A* transcripts from the 630*erm*::P*tet*Spo0A strain grown with increasing concentrations of ATc for 10 h. (B) Western blots of protein extracts from the induced cultures. Coomassie-stained gel is provided as a loading control.

In C. difficile, we have previously shown that the *sin* locus mutant is asporogenic and this phenotype is associated with downregulation of *spo0A* expression. Interestingly, disruption of *sinI*, the second gene in the locus, resulted in elevated levels of sporulation. This result suggested that SinR is a positive regulator of sporulation. Gene expression data from *spo0A* mutants from different studies have shown elevated levels of *sin* locus expression compared to their respective parents (25, 28, 29). These observations taken together suggest that these two master regulators, Spo0A and SinR, regulate each other’s transcription. To understand the possible regulatory relationship between SinR and Spo0A in C. difficile, we created *spo0A* mutants in two different C. difficile strains, JIR8094 and UK1, using the ClosTron mutagenesis technique. Mutation in *spo0A* was confirmed by PCR (see Fig. S1 in the supplemental material) and Western blot analysis using Spo0A-specific antibodies (Fig. 1B). The *spo0A* R20291 mutant, obtained from the Dena Lyras lab (30), was also included in the study. As previously reported, the mutation in *spo0A* resulted in the asporogenic phenotype (24, 25, 30, 31). For complementation, plasmid pRG312 carrying *spo0A* under its own promoter was introduced into the mutants. Heat-resistant spores were observed in the complemented strains; however, the levels were significantly lower than those in the wild type (see Fig. S2 in the supplemental material). To test whether Spo0A influences the expression of the *sin* locus genes, we performed quantitative reverse transcription-PCR (qRT-PCR) analysis of the *sinR* and *sinI* transcripts in *spo0A* mutants and complemented strains, as well as among their respective parent strains. As previously reported, the levels of *sinR* and *sinI* transcripts were increased several-fold (Fig. 1A) in all three *spo0A* mutants compared to their parent strains. An approximately 2-fold reduction in the *sinR* and *sinI* transcripts could be observed in the complemented strains. To further confirm this result, we performed Western blot analysis using SinR-specific antibodies. We grew the mutants and the respective parent strains in TY (tryptose and yeast extract) medium for 10 h and observed the levels of SinR in their cytosol. We found that *spo0A* mutants of all three strains produced larger amounts of SinR compared to their respective parents (Fig. 1B). However, in our complementation of JIR8094::*spo0A* and UK1::*spo0A*, we observed a partial reduction of SinR levels (Fig. 1B). Reduction in SinR level was not obvious in the R20291::*spo0A* complemented strain. Failures to complement an *spo0A* mutation had been previously observed in C. difficile. Two independent studies showed incomplete restoration of the sporulation phenotype in R20291::*spo0A* (28, 30). However, when Deakin et al. tested the *spo0A* mutants of 630Δ*erm* and R20291 strains, they found *in vitro* levels of sporulation to be restored to wild-type levels in their complemented derivative (31). When they tested the R20291 strains for toxin production, however, the complemented strain still produced increased toxin levels compared to the wild type (31). These observations suggest that introducing *spo0A* using a multicopy plasmid may not be a suitable method for complementation considering the Spo0A regulatory networks’ complex nature. In a recent study, Dembek et al. successfully placed P*tet* regulatory elements upstream of the *spo0A* gene, generating 630*erm*::P*tet*Spo0A. This strain can be artificially induced to sporulate by adding anhydrotetracycline (ATc) (32). We obtained this strain and performed SinR Western blotting upon induction of Spo0A. A reduction in *sinR* and *sinI* transcription levels could be seen as Spo0A production increases (Fig. 2A). This observation was further confirmed by Western blot analysis of the Spo0A-induced cultures with SinR-specific antibodies. These results together suggest Spo0A as a negative regulator of the *sin* locus (Fig. 2B).

10.1128/mSphere.00963-20.2 FIG S1 Construction and confirmation of the *spo0A* mutant in the C. difficile JIR8094 and UK1 strains. (A) Schematic representation of ClostTron (group II intron)-mediated insertional inactivation of the *spo0A* gene in C. difficile. (B) PCR verification of the intron insertion and complementation of *spo0A* in JIR8094 with the intron-specific EBS universal primer [EBS(U)] and with *spo0A*-specific primers ORG 551 and ORG 552. (C) PCR verification of the intron insertion in the UK1 strain with EBS(U), ORG 551, and ORG 552. Download FIG S1, TIF file, 1.1 MB. Copyright © 2020 Dhungel and Govind. 2020 Dhungel and Govind This content is distributed under the terms of the Creative Commons Attribution 4.0 International license.

10.1128/mSphere.00963-20.3 FIG S2 Sporulation in *spo0A* mutants. Shown is the percentage of sporulation (the CFU/ml from ethanol-resistant spores) of the parent strain and *spo0A* mutants. The representative results from three independent experiments are shown. Download FIG S2, TIF file, 0.1 MB. Copyright © 2020 Dhungel and Govind. 2020 Dhungel and Govind This content is distributed under the terms of the Creative Commons Attribution 4.0 International license.

### Spo0A represses *sin* locus expression.

**FIG 3:**
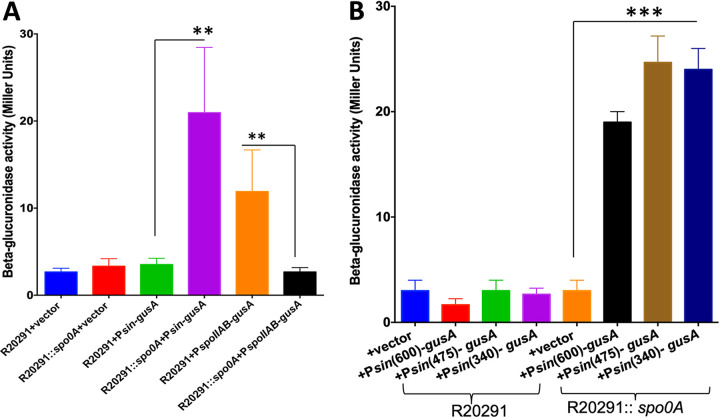
Spo0A represses *sin* locus expression. (A) β-Glucuronidase activity of the P*sin*-*gusA* fusions in the parent strain R20291 and R20291::*spo0A* mutant. Plasmid pBA038 has *gusA* as the reporter gene fused to 600 bp of *sin* locus upstream. A plasmid carrying P*spoIIAB-gusA* (pBA029) and a plasmid carrying a promoterless *gusA* gene (pBA040) were used as positive and negative controls, respectively. (B) Expression of β-glucuronidase in parent strain R20291 and the *spo0A* mutant carrying plasmids pBA037 (475-bp P*sin-gusA*), pBA038 (600-bp P*sin-gusA*), and pBA009 (340-bp P*sin-gusA*). The error bars in panels A and B correspond to standard errors of the means of results from 3 biological replicates, where ** and *** indicate *P* < 0.05 and *P* < 0.005, respectively (by two-tailed Student's *t* test). At least three independent experiments were performed.

Spo0A is a transcriptional regulator and is a DNA binding protein. Spo0A binds to specific DNA sequences in the promoter region of its target gene to regulate their expression. To determine if the elevated levels of SinR observed in *spo0A* mutants are due to the repressor activity of Spo0A, we performed reporter fusion assays. We fused 600 bp of the *sin* locus upstream DNA with the *gusA* reporter gene coding for β-glucuronidase, and the construct was introduced into the R2091::*spo0A* mutant and its parent strain. The plasmid carrying a promoterless *gusA* gene was used as a negative control. We also cloned the promoter region of *spoIIAB*, known to be regulated by Spo0A, with the *gusA* gene and used this construct as a positive control. The *spoIIAB* promoter is positively regulated by Spo0A and was found to be active only in the parent strain and not in the *spo0A* mutant (Fig. 3A). We observed significantly higher β-glucuronidase activity when it was expressed from the *sin* locus promoter in the R20291::*spo0A* mutant strain compared to the parent strain, where very minimal reporter activity was recorded. This observation is consistent with our Western blot results, where we detected elevated levels of SinR in *spo0A* mutant strains. Taken together, these results suggest that Spo0A represses the transcription of *sinR* either directly or indirectly. To narrow down the Spo0A-controlled region in the *sin* locus promoter, we cloned 475 and 340 bp of the upstream DNA with the *gusA* gene and performed the reporter fusion assays. The levels of reporter gene activity were similar in the cultures carrying the 600- and 340-bp upstream fusions (Fig. 3B). This result indicates that both the *sin* locus promoter and the Spo0A-regulated regions are present within this 340-bp region.

### Spo0A binds to the *sin* locus upstream region.

**FIG 4:**
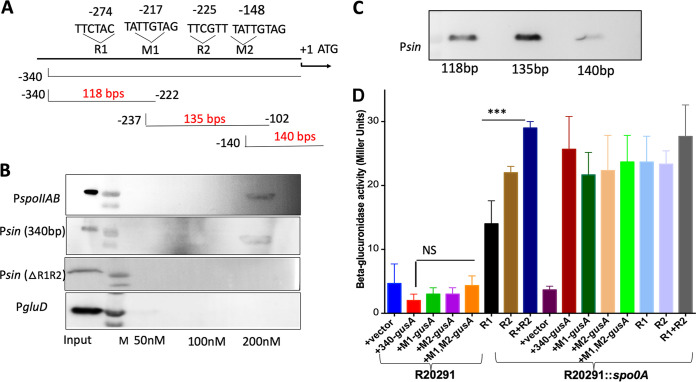
(A) Spo0A binds to *sin* locus upstream DNA. Shown are schematics of the 340-bp upstream *sin* locus denoting the general locations of the M1, M2, R1, and R2 sequences (not to scale) with respect to the translation start (+1) of *sinR*. The lower lines indicate the location and the sizes of the DNA fragments used for the biotinylated-DNA pulldown assay. (B) Western blot analysis using Spo0A-specific antibody to detect endogenous Spo0A in input and eluate fractions. For the biotin-labeled DNA pulldown assay, the promoter regions of *spoIIAB* and *gluD* were used as positive and negative controls, respectively. (C) Three DNA fragments (118, 135, and 140 bp) spanning different regions of the 340-bp *sin* locus upstream were used independently to carry out the binding, and Spo0A was detected as in panel B. (D) Expression of β-glucuronidase in parent strain R20291 and R20291::*spo0A* mutant strains carrying plasmids with *gusA* as the reporter gene fused to the wild-type and mutated promoters of *sinR*. A strain carrying a promoterless *gusA* plasmid (pBA040) was used as a control. Data represent the means ± standard errors of the means (*n *= 3). The asterisks (***) in panel A indicate statistical difference at *P* < 0.005 (by two-tailed Student's *t* test). NS, not significant.

The results in Fig. 3A and B show that expression of P*sin-gusA* was lower in the R20291 background, while the expression of the reporter gene was at higher levels in the R20291::*spo0A* background. To determine whether the repression of *sinR* by Spo0A is due to Spo0A binding specifically to the promoter region of *sinR*, we carried out a DNA binding experiment. Considering that Spo0A needs to be phosphorylated to bind to the target DNA, we did not attempt the *in vitro* electrophoretic gel shift assay. Instead, we used a biotin-labeled DNA pulldown assay to determine the DNA binding ability of Spo0A under native conditions. The DNA segment representing the promoter region of *sinR* was biotinylated and was coupled to immobilized monomeric avidin resin. This bead-DNA complex was incubated with the cell lysate from the parent R20291 strain. The bound proteins were eluted, run in SDS-PAGE, and immunoblotted with the Spo0A antibody. We first standardized the binding experiment by using the *spo0IIAB* promoter region as a positive control. Spo0A protein could be detected in the eluates when the *spo0IIAB* upstream DNA was used as the bait. The biotinylated *gluD* upstream DNA and the beads alone were also processed similarly and served as negative controls. We applied the same protocol using the biotinylated 340-bp *sin* upstream DNA as bait. The results showed that it could pull down Spo0A, suggesting that Spo0A binds specifically to the promoter region of the *sin* locus (Fig. 4A and B). Next, to narrow down the Spo0A binding site within that 340 bp, we created three biotin-labeled fragments covering the first 118 bp (340 to 222 bp upstream), the last 140 bp, and the overlapping 135-bp midregion (237 to 102 bp upstream) (Fig. 4A and C) and used them as bait in the pulldown experiment. Spo0A was detected when the 140-bp midregion and the first 118 bp were used as baits (Fig. 4C). Since the biotin-labeled DNA pulldown assay is semiquantitative, this assumption needs further validation. Spo0A could not be recovered from the eluate from the binding of the *gluD* upstream region or with beads alone (not shown), suggesting the specificity of the Spo0A binding with the *sin* locus promoter.

### Mutational analysis of the *sinR* upstream region.

In B. subtilis, Spo0A∼P is known to bind to the 7-bp DNA element 5′-TGNCGAA-3′, commonly known as the Spo0A box (33). However, there are certain exceptions in which Spo0A binds to degenerated Spo0A boxes with mismatches in the upstream region of some targets (34, 35). The DNA binding domain of C. difficile Spo0A is highly homologous to B. subtilis Spo0A, and the key residues of Spo0A known to mediate the interaction with the bases of the 0A box are highly conserved in *Bacillus* and *Clostridium* species (24, 36). In C. difficile, Spo0A is known to bind to *spo0A* upstream and *sigH* upstream. Both of these genes have the TGTCGAA consensus Spo0A box sequence (23, 37). C. difficile Spo0A also binds upstream of an *spoIIAA*-*spoIIE*-*spoIIGA* operon with low affinity, where the binding sequence is a degenerated Spo0A box with TACGACA sequence (23). We scanned the upstream region of *sinR* for potential Spo0A binding consensus sequence. We could predict that Spo0A binds to sequences within the 340 to 102 bp upstream of the *sin* locus from the biotin pulldown experiment. A classical Spo0A binding box (TTCTACA, complementary to TGTAGAA [marked as R1]) could be identified 274 bp upstream of the start codon. A potential degenerated Spo0A box (TTCGTTT [marked as R2]) was located 230 bp upstream. Two repeats with TATTGTAG sequences could also be seen in this region. We included all four regions for further analysis. We mutated the C and G residues in these chosen regions with T, and these mutated *sin* upstream regions were used to create reporter fusions. Mutations in the repeat sequences didn’t affect the expression of the reported genes. However, mutations in the predicted Spo0A boxes (R1 and R2) affected the expression of the reporter fusion (Fig. 4D). These mutations relieved the repression observed in the parent strain, and the reporter activity was similar to the one observed in the *spo0A* mutant. To further confirm this result, we performed a biotin pulldown assay with the *sin* locus upstream DNA carrying both the R1 and R2 mutations. Spo0A couldn’t be pulled down when this mutated DNA fragment was used as a bait (Fig. 4B). These results demonstrate that Spo0A binds to the R1 and R2 regions in the *sin* locus upstream to repress its expression.

## DISCUSSION

Sporulation in a cell is an intense response to stress and is particularly expensive in both time and materials (38). The exact conditions and timing for sporulation are likely to be under strong selective pressure as both premature spore production and belated production can have disastrous effects on cell growth and survival. In B. subtilis, the *sin* (*s*porulation *in*hibition) operon is central to the timing and early dynamics of this network (39–41), and its regulation is controlled by the sporulation master regulator Spo0A itself. In this study, we have demonstrated that similar to B. subtilis, the *sin* locus in C. difficile is also regulated by Spo0A.

Like in many Gram-positive bacteria, Spo0A is the master regulator of sporulation in C. difficile (24, 30, 31). When the post-exponential phase begins, Spo0A activates the expression of the genes involved in the sporulation initiation process and positively regulates the sigma factor cascade required for sporulation (29). In many other pathogenic spore-forming bacteria, the gene regulatory networks that influence sporulation and virulence are closely linked with each other (42–46). In C. difficile, the mutation in *spo0A* affected many pathogenic traits, including toxin production, flagellum expression, and biofilm formation (5, 28, 30, 31, 47). Mackin et al. observed a clear increase in the production of toxins A and B upon disruption of *spo0A* in the ribotype 027 isolates R20291 and M7404 (30). In a similar study, Deakin et al. found that an R20291 *spo0A* mutant caused more severe disease in a murine model than the wild-type strain and associated this increase in severity with an increase in the amount of toxins A and B produced by the mutant *in vitro* (31). Dawson et al. showed that Spo0A in the 630Δ*erm* strain promotes a sporulation cascade and biofilm formation and negatively regulates expression of virulence factors (toxins and flagella) (28). We found the UK1::*spo0A* strain to produce higher levels of toxins than its parent strain, while no significant difference was observed between the JIR8094 parent and JIR8094::*spo0A* mutant (see Fig. S3A in the supplemental material). This observation was consistent with the previous report, where a mutation in *spo0A* influenced the toxin production only in the 027 ribotype, which includes the UK1 and R20291 strains, but not in the 630Δ*erm* strain, which belongs to the 012 ribotype like the JIR8094 strain. Reduced biofilm formation was also found only in R20291::*spo0A* and UK1::*spo0A* (Fig. S3B and C). The mechanism of Spo0A regulation over these pathways remains to be answered. In the C. difficile genome, >100 open reading frames have potential 0A boxes within 500 bp of their start codons, indicating direct regulation by Spo0A (24). However, *tcdA* and *tcdB*, encoding toxins A and B, respectively, are not among them, indicating the indirect influence of Spo0A on toxin production (24). Spo0A could indirectly control motility and biofilm formation since many candidate regulators are encoded by the genes putatively under the direct control of Spo0A in C. difficile (24, 31). Our current finding of Spo0A-mediated *sin* locus regulation can partly explain many of the phenotypes displayed by *spo0A* mutants, especially in the ribotype 027 strains (28, 30). In our initial characterization of the *sin* locus, we showed decreased toxin production and motility in the absence of SinR and SinI (15). The expression of *sinR* alone was sufficient to complement these phenotypes and suggested SinR as a positive regulator of these pathways (15). We have further shown that SinR controls toxin production by regulating *sigD*, a sigma factor that positively regulates *tcdR*, which is needed for the transcription of toxin genes (15, 48, 49). SigD is also needed for the transcription of the flagellar operon in C. difficile (48, 49). This study has shown increased SinR production in the absence of Spo0A (Fig. 1A and B). qRT-PCR results showed increased expression of *sigD*, *tcdR*, and *tcdB* in the R20291::*spo0A* and UK1::*spo0A* strains compared to their respective parent strains (Fig. S3C). Increased *sigD* expression can lead to increased flagellar and toxin production and reduced biofilm formation in the *spo0A* mutant (22, 28) (Fig. S3).

10.1128/mSphere.00963-20.4 FIG S3 Toxin gene transcription and biofilm formation in the R20291::*spo0A* mutant strain. (A) Toxin production measured by ELISA. Statistical analysis was performed using Student’s *t* test. (**, *P* < 0.05). (B) Crystal violet-stained biofilm in the 12-well tissue culture plate, showing poor biofilm formation in the R20291::*spo0A* and UK1::*spo0A* mutants. (C) Relative expression of the transcripts of *tcdR*, *tcdB*, and *sigD* genes from C. difficile. RNA was collected from the parent and *spo0A* mutant strains in the JIR8094, R20291, and UK1 backgrounds at the 10-h time point. Download FIG S3, TIF file, 0.2 MB. Copyright © 2020 Dhungel and Govind. 2020 Dhungel and Govind This content is distributed under the terms of the Creative Commons Attribution 4.0 International license.

In this study, we have shown that Spo0A binds to the C. difficile *sin* locus promoter and suppresses the expression of both *sinR* and *sinI*. We had previously shown that disruption of *sinR* by insertion mutagenesis affects transcription of both *sinR* and *sinI* (15), suggesting that *sinRI* is transcribed as a bicistronic message. Our qRT-PCR analysis detected lower levels of *sinI* transcripts than *sinR* transcripts. Since the reduction is observed in both the parent strain and *spo0A* mutants, we can conclude that this effect is independent of Spo0A.

In summary, we have demonstrated that Spo0A, the master regulator of sporulation, regulates *sin* locus expression. We have further shown that Spo0A can bind to the upstream region of the *sin* locus and have successfully mapped the region to which it binds. This finding adds a new detail to C. difficile’s virulence gene regulatory network.

## MATERIALS AND METHODS

### Bacterial strains and growth conditions.

C. difficile strains (see Table S1 in the supplemental material) were grown in TY (tryptose and yeast extract) agar or broth culture in an anaerobic chamber maintained at 10% H_2_, 10% CO_2_, and 80% N_2_ as described previously (27, 50–52). Lincomycin (Lin; 20 μg/ml) and thiamphenicol (Thio; 15 μg/ml) were added to the culture medium when required. S17-1, an Escherichia coli strain used for conjugation (53), was cultured aerobically in LB (Luria-Bertani) broth or agar and was supplemented with ampicillin (100 μg/ml) or chloramphenicol (25 μg/ml) when necessary.

10.1128/mSphere.00963-20.5 TABLE S1 Bacterial strains and plasmids used in this study. Download Table S1, DOCX file, 0.02 MB. Copyright © 2020 Dhungel and Govind. 2020 Dhungel and Govind This content is distributed under the terms of the Creative Commons Attribution 4.0 International license.

### General DNA techniques.

Chromosomal DNA was extracted from C. difficile cultures with the DNeasy blood and tissue kit (Qiagen). PCRs were carried out using gene-specific primers (see Table S2 in the supplemental material). PCR products were extracted from the gel with the Geneclean kit (mpbio). Plasmid DNA was extracted using the QIAprep spin miniprep kit (Qiagen). Standard procedures were used to perform routine cloning.

10.1128/mSphere.00963-20.6 TABLE S2 Oligonucleotides used in the study. Download Table S2, DOCX file, 0.02 MB. Copyright © 2020 Dhungel and Govind. 2020 Dhungel and Govind This content is distributed under the terms of the Creative Commons Attribution 4.0 International license.

### Construction and complementation of C. difficile *spo0A* mutant strains.

The *spo0A* mutants of the JIR8094 and UK1 strains were created using the ClosTron gene knockout system as described previously (24, 25, 28, 31). Briefly, for *spo0A* disruption, the group II intron insertion site between nucleotides 178 and 179 in the *spo0A* gene in the antisense orientation was selected using a web-based design tool called the Perutka algorithm. The designed retargeted intron was cloned into pMTL007-CE5, as described previously (54). The resulting plasmid, pMTL007-CE5::*spo0A*-178-179a, was transferred into C. difficile UK1 and JIR8094 cells by conjugation. The potential Ll.ltrB insertions within the target genes were conferred by the selection of lincomycin-resistant transconjugants in 20-μg/ml lincomycin plates. PCR using gene-specific primers (Table S2) in combination with the EBS(U) universal primer was performed to identify putative C. difficile mutants. C. difficile *spo0A* mutants were complemented by introducing pRG312, which contains the *spo0A* gene with the 300-bp upstream region, through conjugation. Complementation was confirmed by PCR and Western blot analysis.

### Western blot analysis.

C. difficile cultures for Western blot analysis were harvested and washed in 1× phosphate-buffered saline (PBS) solution. The pellets were resuspended in sample buffer (80 mM Tris, 2% SDS, 10% glycerol) and lysed by sonication. The whole-cell extracts were then centrifuged at 17,000 × *g* at 4°C for 1 min. The lysate was heated at 100°C for 7 min, and the proteins were separated by SDS-PAGE and electroblotted onto polyvinylidene difluoride (PVDF) membrane. The blots were then probed with specific primary and secondary antibodies at a dilution of 1:10,000. Immunodetection of proteins was done using the ECL enhanced chemiluminescence kit (Millipore) following the manufacturer’s recommendations and were developed using the G-Box iChemi XR scanner. Blot images were overlapped with the original images of the membrane to visualize the prestained marker.

### Construction of reporter plasmids and β-glucuronidase assay.

The *sin* locus upstream DNA regions of various lengths were amplified by PCR using specific primers with KpnI and SacI (Table S2) recognition sequences. R20291 strain chromosomal DNA was used as a template for this amplification. Plasmid pRPF185 carries a *gusA* gene for β-glucuronidase under a tetracycline-inducible (*tet*) promoter. The *tet* promoter was removed using KpnI and SacI digestion and was replaced with *sin* locus upstream regions of various lengths to create plasmids pBA009, pBA029, pBA037, pBA038, and pBA039 (Table S1). The control plasmid pBA040 with a promoterless *gusA* gene was created by digestion with KpnI and SacI to remove the *tet* promoter and then self-ligated after creating blunt ends. Plasmids were introduced into the R20291 and R20291::*spo0A* strains through conjugation as described previously (15, 27). The transconjugants were grown in TY medium in the presence of thiamphenicol (15 μg/ml) overnight. These overnight cultures were then used as an inoculum at a 1:100 dilution to start a new culture. Bacterial cultures were harvested at 10 h of growth, and the amount of β-glucuronidase activity was assessed as described elsewhere (55, 56).

### Mutagenesis of *sin* locus promoter region.

A Quick Change Lightning site-directed mutagenesis kit (Agilent Technologies) was used to carry out site-directed mutagenesis whereby G and C residues of the potential Spo0A binding 0A boxes were substituted for with A residues. The mutagenic oligonucleotide primers used are listed in Table S2. Synthetic DNA fragments with R1 and R2 mutations (Fig. 4A) were delivered cloned into pUC57 by Genewiz, which were later used to create reporter fusions and for the biotin pulldown assays.

### Biotin pulldown assays.

Biotin pulldown assays were carried out as described elsewhere (57). Briefly, the P*sinR* DNA fragment was biotin labeled and was coupled to immobilized monomeric avidin resin (G Biosciences) in B/W buffer (57). P*gluD* (upstream of *gluD* coding for glutamate dehydrogenase) and bead-alone negative controls were treated alongside test samples. The DNA and the beads were incubated at room temperature for 30 min in a rotor. The bead-DNA complex was washed with TE buffer to remove any unbound DNA. To prepare cell lysates, C. difficile R20291 strain was grown to the late exponential phase (16 h) in 500 ml TY medium at pH 7.4. After washing with 1× PBS, the cells were resuspended in BS/THES buffer (57) and lysed using a French press. The whole lysate was centrifuged at 20,000 × *g* for 30 min at 4°C. The supernatant, along with salmon sperm DNA as a nonspecific competitor, was incubated with the bead-DNA complex and allowed to rotate at 4°C overnight. The bead-DNA-protein complex was washed with BS/THES buffer (5 times). Elution was carried out with 50, 100, and 200 mM NaCl in Tris-HCl at pH 7.4. The eluates were analyzed by SDS-PAGE and Western blotting using Spo0A-specific antibody.

10.1128/mSphere.00963-20.1 TEXT S1 Supplemental methods: sporulation assay, toxin assays, qRT-PCR, and biofilm assay. Download Text S1, DOCX file, 0.02 MB. Copyright © 2020 Dhungel and Govind. 2020 Dhungel and Govind This content is distributed under the terms of the Creative Commons Attribution 4.0 International license.

10.1128/mSphere.00963-20.7 TABLE S3 Oligonucleotides used for qRT-PCR. Download Table S3, DOCX file, 0.01 MB. Copyright © 2020 Dhungel and Govind. 2020 Dhungel and Govind This content is distributed under the terms of the Creative Commons Attribution 4.0 International license.
