# Supplementary figures and images for: Spo0A Suppresses sin Locus Expression in Clostridioides difficile
Source: mSphere. 2020 Nov 4;5(6):e00963-20. doi: 10.1128/mSphere.00963-20 (PMC7643835; doi:10.1128/mSphere.00963-20)

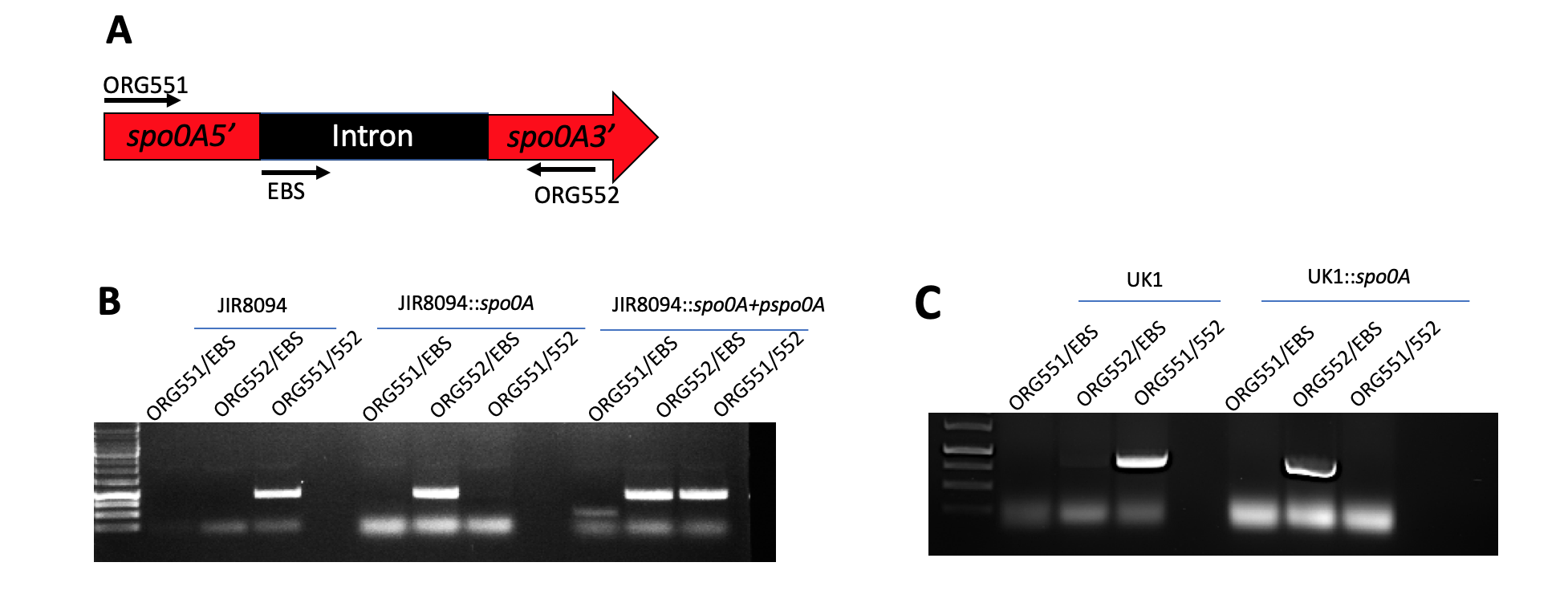

Supplement: FIG S1 [file mSphere.00963-20-sf001.tif]

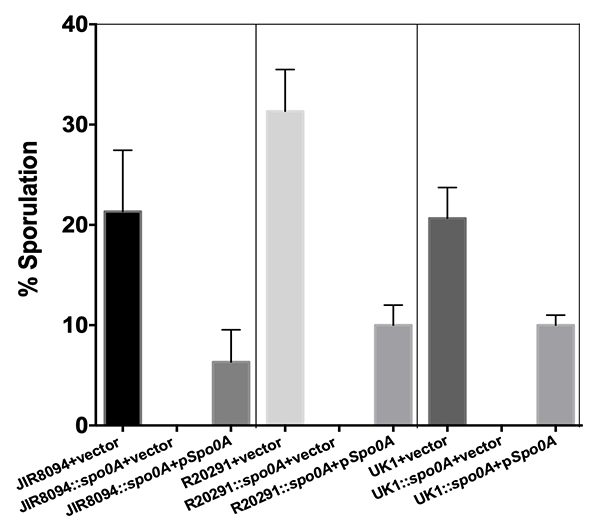

Supplement: FIG S2 [file mSphere.00963-20-sf002.tif]

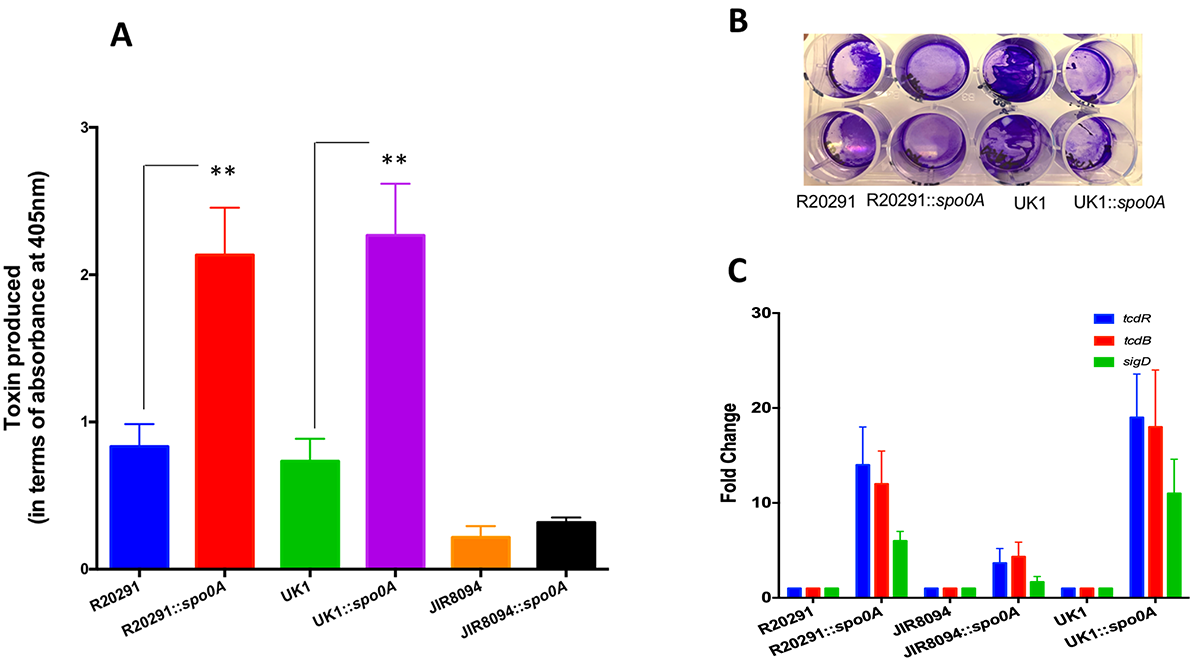

Supplement: FIG S3 [file mSphere.00963-20-sf003.tif]
