# Supplementary material for: Spo0A Suppresses sin Locus Expression in Clostridioides difficile
Source: mSphere. 2020 Nov 4;5(6):e00963-20. doi: 10.1128/mSphere.00963-20 (PMC7643835; doi:10.1128/mSphere.00963-20)
Supplement: TABLE S1 [file mSphere.00963-20-st001.docx]

**S1. Table: Bacterial strains and plasmids used in this study**

| **Bacterial strain or plasmid** | **Relevant features or genotype** | **Source or reference** |
| --- | --- | --- |
| *Clostridioides difficile* JIR8094 | Erm^s^ derivative of strain 630 | O’Connor *et al.* (2006) |
| *C. difficile* R20291 | Clinical isolate - NAP1/027 ribotype, isolated in 2006 following an outbreak in Stoke Mandeville Hospital, UK | Stabler *et al.* (2009) |
| *C. difficile* UK1 | Clinical isolate | Sorg *et al*., (2010) |
| *Escherichia coli* DH5α | *endA1 recA1 deoR hsdR17 (r*_K_^−^ *m*_K_^+^*)* | NEB |
| *E. coli* S17-1 | Strain with integrated RP4 conjugation transfer function; favors conjugation between *E. coli* and *C. difficile* | Teng *et al.* (1998) |
| *C. difficile* JIR8094::*spo0A* | JIR8094 with intron insertion within *spo0A* | This study |
| *C. difficile* R20291::*spo0A* | R20291with intron insertion within *spo0A* | Mackin *et al.* (2013) |
| *C. difficile UK1::spo0A* | UK1 with intron insertion within *spo0A* | This study |
| *C. difficile 630erm::PtetSpo0A* | 630erm strain with *spo0A* under *tet* inducible promoter | Dembek et al. (2017) |
| pMTL007-CE5 | ClosTron plasmid | Heap *et al.* (2010) |
| pMTL007-CE5:Cdi-*spo0A*-178-179a | pMTL007-CE5 with group II intron targeted to *spo0A* | This study |
| pRG312 | pMTL84151 containing 300bp upstream *spo0A* with *spo0A* gene | Girinathan *et al.* (2018) |
| pRPF185 | *E. coli*/ *C. difficile* shuttle plasmid | Fagan *et al*. (2011) |
| pBA009 | pRPF185 with 340 bp P*sin-gusA* | This study |
| pBA029 | pRPF185 with P*spoIIAB-gusA* | This study |
| pBA030 | pRPF185 with 340 bp P*sin-gusA* with M1 mutation (Fig. 3) | This study |
| pBA031 | pRPF185 with 340 bp P*sin-gusA* with M2 mutation (Fig. 3) | This study |
| pBA039 | pRPF185 with 340 bp P*sin-gusA* with M1& M2 (Fig. 3) | This study |
| pBA038 | pRPF185 with 600 bp P*sin-gusA* (Fig.1) | This study |
| pBA037 | pRPF185 with 475 bp P*sin-* *gusA* (Fig. 1) | This study |
| pBA040 | pRPF185 with a promoter less *gusA* | This study |
| pBA041 | pRPF185 with 340 bp P*sin-gusA* with R1 mutation (Fig. 3) | This study |
| pBA042 | pRPF185 with 340 bp P*sin-gusA* with R2 mutation (Fig. 3) | This study |
| pBA043 | pRPF185 with 340 bp P*sin-gusA* with R1&R2 mutation (Fig. 3) | This study |

**References:**

1. O'Connor JR, Lyras D, Farrow KA, Adams V, Powell DR, Hinds J, et al. Construction and analysis of chromosomal *Clostridium difficile* mutants. Mol Microbiol. 2006;61(5):1335-51. doi: 10.1111/j.1365-2958.2006.05315.x. PMID: 16925561.
2. Stabler RA, He M, Dawson L, Martin M, Valiente E, Corton C, et al. Comparative genome and phenotypic analysis of *Clostridium difficile* 027 strains provides insight into the evolution of a hypervirulent bacterium. Genome Biol. 2009;10(9):R102. doi: 10.1186/gb-2009-10-9-r102. PMID: 19781061.
3. Teng F, Murray BE, Weinstock GM. Conjugal transfer of plasmid DNA from *Escherichia coli* to enterococci: a method to make insertion mutations. Plasmid. 1998;39(3):182-6. doi: 10.1006/plas.1998.1336. PMID: 9571134.
4. Sorg JA, Sonenshein AL. Inhibiting the initiation of *Clostridium difficile* spore germination using analogs of chenodeoxycholic acid, a bile acid. J Bacteriol. 2010;192(19):4983-90. doi: 10.1128/JB.00610-10. PMID: 20675492.
5. Heap JT, Kuehne SA, Ehsaan M, Cartman ST, Cooksley CM, Scott JC, et al. The ClosTron: Mutagenesis in *Clostridium* refined and streamlined. J Microbiol Methods. 2010;80(1):49-55. doi: 10.1016/j.mimet.2009.10.018. PMID: 19891996.
6. Fagan RP, Fairweather NF. *Clostridium difficile* has two parallel and essential Sec secretion systems. J Biol Chem. 2011;286(31):27483-93. doi: 10.1074/jbc.M111.263889. PMID: 21659510.
7. [Mackin KE](https://www.ncbi.nlm.nih.gov/pubmed/?term=Mackin%20KE%5BAuthor%5D&cauthor=true&cauthor_uid=24236153), [Carter GP](https://www.ncbi.nlm.nih.gov/pubmed/?term=Carter%20GP%5BAuthor%5D&cauthor=true&cauthor_uid=24236153) [Howarth P](https://www.ncbi.nlm.nih.gov/pubmed/?term=Howarth%20P%5BAuthor%5D&cauthor=true&cauthor_uid=24236153), [Rood JI](https://www.ncbi.nlm.nih.gov/pubmed/?term=Rood%20JI%5BAuthor%5D&cauthor=true&cauthor_uid=24236153), [Lyras D](https://www.ncbi.nlm.nih.gov/pubmed/?term=Lyras%20D%5BAuthor%5D&cauthor=true&cauthor_uid=24236153). Spo0A Differentially Regulates Toxin Production in Evolutionarily Diverse Strains of *Clostridium difficile.* Plos One. 2013; 8(11):e79666. <https://doi.org/10.1371/journal.pone.0079666>.
8. Dembek, Marcin, Stephanie E. Willing, Huynh A. Hong, Siamand Hosseini, Paula S. Salgado, and Simon M. Cutting. “Inducible Expression of Spo0A as a Universal Tool for Studying Sporulation in *Clostridium Difficile*.” *Frontiers in Microbiology* 8 (September 21, 2017). https://doi.org/10.3389/fmicb.2017.01793.
