# Supplementary material for: Spo0A Suppresses sin Locus Expression in Clostridioides difficile
Source: mSphere. 2020 Nov 4;5(6):e00963-20. doi: 10.1128/mSphere.00963-20 (PMC7643835; doi:10.1128/mSphere.00963-20)
Supplement: TABLE S2 [file mSphere.00963-20-st002.docx]

**S2. Table**

**Oligonucleotides used in this study**

| Name | Sequence (5’ 🡪 3’) | Description |
| --- | --- | --- |
| EBS-U | CGA AAT TAG AAA CTT GCG TTC AGT AAAC | Group II intron specific primer |
| ORG749 | GGTACCATTTACATAAATTGTTCAATATATAAAATAGAAAA | *sin* locus upstream (340 bps) to clone in pRPF185 (Forward with KpnI) |
| ORG750 | GAGCTCAATTATTATCCCTCCACTTTAGATTATATTCAT | *sin* locus upstream (340 bps) to cline in PRPF185 (Reverse with SacI) |
| ORG809 | 5Biosg/TTTGGAAGCATTGATAGAATAAATAAAATCAATATAGAG | Forward 350 pb upstream GluD (GDH) R20291_5’ biotin |
| ORG810 | 5Biosg/ TTTGAAAGCCCCCTTATAAATACGTTATAATTATGTATACTCC | Reverse 350 pb upstream GluD (GDH) R20291_5’ biotin |
| ORG811 | 5Biosg/ATTTACATAAATTGTTCAATATATAAAATAGAAAA | Forward 340 bp upstream *sinR* 5’ biotin labelled |
| ORG812 | 5Biosg/AATTATTATCCCTCCACTTTAGATTATATTCAT | Reverse 340 bp upstream *sinR* 5’ biotin labelled |
| ORG825 | GGTACCGTAGGTAATTATATAGTAAAAATGTATGTTGGTTGGTGGAAGAAACATGG | *spoIIAB* upstream Forward with KpnI |
| ORG826 | GAGCTCCAAAAATCCCTCCTTCAATAGTTTTGTAAAAATAGTAAC | *spoIIAB* upstream Reverse with SacI |
| ORG 827 | AGAAATATCTTCATAATTTCATAGCCATATTATAATAATAAAACGAATTAATAATATGTTAATTATAGCATTTTTTATTGTAATTACA | 340 bp upstream sin M1 mutagenesis Forward |
| ORG 828 | TGTAATTACAATAAAAAATGCTATAATTAACATATTATTAATTCGTTTTATTATTATAATATGGCTATGAAATTATGAAGATATTTCT | 340 bp upstream sin M1 mutagenesis Reverse |
| ORG 829 | GTGCTATTTATTTTTGTATAGTCTGTATTTATAATAAACAAATATGGATAAATCATATTTTTAATTAGAGAAATATCTTCATAAT | 340 bp upstream sin M2 mutagenesis Forward |
| ORG 830 | ATTATGAAGATATTTCTCTAATTAAAAATATGATTTATCCATATTTGTTTATTATAAATACAGACTATACAAAAATAAATAGCAC | 340 bp upstream sin M2 mutagenesis Reverse |
| ORG 850 | 5/Biosg/GTAGGTAATTATATAGTAAAAATGTATGTTGGTTGGTGGAAGAAACATGG | *spoIIAB* promoter_5’ biotinylated _Forward |
| ORG 851 | 5/Biosg/CAAAAATCCCTCCTTCAATAGTTTTGTAAAAATAGTAAC | *spoIIAB* promoter_5’ biotinylated _Reverse |
| ORG 873 | 5/Biosg/GCACTTATTTCAGTATAAAAACATATATAGTCTAT | *sin* locus 340 bps upstream (Forward to amplify @ last 140 bps) |
| ORG 872 | 5/Biosg/CTGAAATAAGTGCTATTTATTTTTGTATAGTCTGTATC | *sin* locus 340 bps upstream (Reverse to amplify @ middle 135 bps) |
| ORG 871 | 5/Biosg/CATATTATTAATTCGTTTTATTATTGTAGTATGGC | *sin* locus 340 bps upstream (Forward to amplify @ middle 135 bps) |
| ORG 870 | 5/Biosg/CGAATTAATAATATGTTAATTATAGCAT | *sin* locus 340 bps upstream (Reverse to amplify @ first 118 bps) |
| ORG 889 | TTTAGGTACCTTAAATTATTTTATAAGATTATTACTCTACTATAAATCTTGTATATAACT | *sin* locus upstream 600 bps Forward with KpnI |
| ORG 890 | TTTAGGTACCTTCTAAATGCCTTACTTATAATTAATTTTTTATTTCACCTATATATAATT | *sin* locus upstream 475 bps Forward with KpnI |
