# Supplementary material for: Spo0A Suppresses sin Locus Expression in Clostridioides difficile
Source: mSphere. 2020 Nov 4;5(6):e00963-20. doi: 10.1128/mSphere.00963-20 (PMC7643835; doi:10.1128/mSphere.00963-20)
Supplement: TEXT S1 [file mSphere.00963-20-s0001.docx]

**Supplemental Methods:**

**Sporulation assay**

Sporulation assay were performed as described previously (Girinathan *et al.* 2018). Briefly, *C*. *difficile* strains were grown on 70:30 sporulation agar. After 30 h of growth, cells were scraped from the plates and suspended in 70:30 sporulation liquid medium to an OD_600_ of 1.0. To enumerate viable vegetative cells and spores, cells were serially diluted and plated onto TY agar with 0.1% taurocholate and incubated at 37^°^C for 24 to 48 hours. To enumerate the number of viable spores only, 500μl of the samples from each culture were mixed 1:1 with 95% ethanol and incubated for 1hour at room temperature to kill all the vegetative cells. The ethanol-treated samples were then serially diluted, plated on TY agar with 0.1% taurocholate and incubated at 37^°^C for 24 to 48 hours. The percentage of ethanol-resistant spores were calculated by dividing the number of CFU from spores by the total number of CFU and multiplying the value by 100. The results were based on a minimum of three biological replicates.

**Quantitative reverse transcription PCR (qRT-PCR)**

*C. difficile* cultures were grown in TY medium for 10h and total RNA was extracted following a previously described protocol (Girinathan *et al.* 2018). Briefly, total RNA was treated with DNAse (Turbo; Ambion) for 2 h at 37^°^C. After DNase treatment, a 25 µL reaction was set up with 1 µg of template RNA and was heated at 70 °C for 10 min. The same reaction was made to 50 ul by adding 10 µL of 5X reverse transcription (RT) reaction buffer, 1mM of dNTP; 1 µg of hexamer oligonucleotide primer (5 µg/µL pdN_6_; Roche), and avian myeloblastosis virus (AMV) reverse transcriptase (Promega) to synthesize cDNA at 42 °C for 2 hours. Real-time quantitative PCR was performed in iQPCR real-time PCR instrument (BioRad) by setting up 20 µL reaction volume containing 10 ng of cDNA, 400 nM gene-specific primers, and 10 µL of SYBR PCR master mix (BioRad). Quantity of cDNA of a gene in each sample was normalized to the quantity of *C. difficile* 16S rRNA gene and the ratio of normalized target concentrations (threshold cycle [2^−ΔΔCt^] method) gives the relative change in gene expression. A minimum of three biological replicates were used per sample.

**Toxin ELISA**

Cytosolic toxins from 12h old C. difficile cultures grown in TY medium were measured as described previously (Girinathan *et al.* 2018). In brief, one ml of C. difficile cultures were harvested and suspended in 200 μl of sterile PBS, sonicated and centrifuged to harvest the cytosolic protein. One hundred μg of cytosolic proteins was used to measure the relative toxin levels using C. difficile premier Toxin A &B ELISA kit from Meridian Diagnostics Inc. (Cincinnati, OH).

**Biofilm Assay**

Biofilm assay was performed as described by Poquet et. al, 2018 with minor modifications. Briefly, *C. difficile* strains grown in TY medium for 16h and were diluted two-fold in fresh TY. Two ml of the diluted over-night cultures were added to 12-well micro-titer plates and was incubated for 3 hr at 37°C. After carefully removing the liquid, fresh TY medium was added onto the adhesive cells and were incubated at 37°C. After 24 hr, the supernatant was removed from the wells, being careful not to touch the bottom of the plate. The biofilms were then washed once with phosphate-buffered saline (PBS), stained for 30 min in 0.1% filtered crystal violet, and washed twice with PBS. To quantify the biofilm formation, 100% ethanol was used to solubilized the dye and quantified at 570 nm.

Reference:

Girinathan BP, Ou J, Dupuy B, Govind R. 2018. Pleiotropic roles of *Clostridium difficile* *sin* locus. PLoS. Pathog 14:e1006940. https://doi.org/10.1371/journal.ppat.1006940.

Poquet I, Saujet L, Canette A, Monot M, Mihajlovic J, Ghigo J-M, Soutourina O, Briandet R, Martin-Verstraete I, Dupuy B. 2018. *Clostridium difficile* biofilm: remodeling metabolism and cell surface to build a sparse and heterogeneously aggregated architecture. Front. Microbiol 9:2084. https://doi.org/10.3389/fmicb.2018.02084.
