# Supplementary material for: Spo0A Suppresses sin Locus Expression in Clostridioides difficile
Source: mSphere. 2020 Nov 4;5(6):e00963-20. doi: 10.1128/mSphere.00963-20 (PMC7643835; doi:10.1128/mSphere.00963-20)
Supplement: TABLE S3 [file mSphere.00963-20-st003.docx]

S3 Table. Oligonucleotides used for QRT-PCR

| Primer | Sequence (5’ 🡪 3’) | Gene target |
| --- | --- | --- |
| RG-RT23 (F) | GAGGAGAGTGGAATTCCTAGTGTAG | *16srRNA* |
| RG-RT24 (R) | GGACTACCAGGGTATCTAATCCTGT | *16srRNA* |
| RG-RT25 (F) | AGGCAGGTTTACATCCAACATA | *sinR* |
| RG-RT26 (R) | AGTGGTATGTCTAAAGCAGTAGC | *sinR* |
| RG-RT27 (F) | AAAGACTTAAAGAAGAACGGAAAA | *sinR’* |
| RG-RT28 (R) | TTGGATTCTTTTTACCACTTTCG | *sinR’* |
| RG-RT7 (F) | CTGGACAATGGAAGGTGGTT | *tcdB* |
| RG-RT8 (R) | TTGATGGTGCTGAAAAGAAGTG | *tcdB* |
| RG-RT1 (F) | CAAGAAATAACTCAGTAGATGATTTGCAA | *tcdR* |
| RG-RT2 (R) | TCTCCCTCTTCATAATGTAAAACTCTACTA | *tcdR* |
| *sigD*-RT(F) | \| TGATAGAGAAGAGGAAGCTCCA \| \| --- \| | *sigD* |
| *sigD*-RT(R) | TCTGAAACACCTAGCACTTTTCC | *sigD* |
| RG-RT33 (F) | CATGAAATAGGAGTACCAGCTCA | *spo0A* |
| RG-RT34 (R) | CTCCATGCAACCTCTATTGC | *spo0A* |
